# The Proliferation of Glioblastoma Is Contributed to Kinesin Family Member 18A and Medical Data Analysis of GBM

**DOI:** 10.3389/fgene.2022.858882

**Published:** 2022-04-08

**Authors:** Lei-Bo Wang, Xue-Bin Zhang, Jun Liu, Qing-Jun Liu

**Affiliations:** ^1^ Department of Neurosurgery, Tianjin Huanhu Hospital, Tianjin, China; ^2^ Department of Pathology, Tianjin Huanhu Hospital, Tianjin, China

**Keywords:** glioblastoma, kinesin family member 18A, therapeutic target, proliferation, medical data analysis

## Abstract

**Background**: Glioblastoma (GBM) is widely known as a classical kind of malignant tumor originating in the brain with high morbidity and mortality. Targeted therapy has shown great promise in treating glioblastoma, but more promising targets, including effective therapeutic targets, remain to be identified. 18A (KIF18A) is a microtubule-based motor protein that is dysregulated and involved in the progression of multiple human cancers. However, the possible effects of KIF18A on GBM progression are still unclear.

**Methods**: We performed DEG analysis, medical data analysis, and network analysis to identify critical genes affecting glioma progression. We also performed immunohistochemical analysis of the KIF18A levels in 94 patients with glioblastoma and the associated surrounding tissues. Patients were divided into two groups according to the high and low expression. Using a clinical analysis, we showed the potential associations between KIF18A expression and clinical characteristics of 94 GBM patients. We then investigated the effects of KIF18A on GBM cell proliferation by colony establishment, MTT, and immune blogging. The possible effect of KIF18A on GBM tumor growth was determined in mice.

**Results**: We identified KIF18A as a potential gene affecting GBM progression. We further demonstrated that GBM tissues expressed KIF18A much higher, and its presentation was associated with recurrence in glioblastoma patients. We believe KIF18A promotes GBM cell proliferation.

**Conclusion**: We demonstrated that KIF18A could be a promising target in treating GBM.

## Introduction

Each year, 23,880 patients are re-diagnosed with brain tumors or other neurological disorders, and approximately 16,830 die from brain-related diseases. Glioblastoma (GBM) is a type of brain astrocytoma. Clinical treatment is made more difficult by aggressive GBM growth (de Paula et al., 2017; Pasqualetti et al., 2018; Siegel et al., 2018; Wachowiak et al., 2018). Treatment of GBM mainly consists of surgery, radiation, and chemotherapy. In some cases, surgical removal of the tumor can lead to recurrence. There is an urgent need for further treatment. Furthermore, immunotherapy and targeted therapies are also promising. The exact mechanisms regulating GBM progression remain to be investigated. It should be emphasized that there is an urgent need for effective treatment goals for GBM.

*KIF18A* is a member of the microtubule-associated wire (KIF) superfamily, which has been shown to influence the progression of various cancers. Actin plays a key role in cellular processes such as cell morphology, cytoskeletal dynamics, intracellular transport of macromolecules/organelles, and cell division. Therefore, actin may be involved in cancer development. *KIF18A* is a form of mononucleosis characterized by monocytosis (mononucleosis) and monocytosis (mononucleosis). *KIF18A* dysregulation can lead to chromosomal instability. Previous studies have shown that KIF18A is overexpressed in many cancers, including renal, breast, and hepatocellular carcinomas (Frisch and Screaton, 2001; Zhu et al., 2005; Mayr et al., 2007; Wagenbach, 2008; Zhang et al., 2010; Häfner et al., 2014; Kim et al., 2014; Braun et al., 2015; Czechanski et al., 2015; Okonogi et al., 2015; Shin et al., 2015; Chen et al., 2016; Kasahara et al., 2016; Wordeman et al., 2016; Möckel et al., 2017; Morgan and Mason, 2017; Pratt et al., 2017; Dai et al., 2018; Fotakopoulos et al., 2018; Jain, 2018; Luo et al., 2018). Furthermore, the reduction of KIF18A significantly induced apoptosis in human breast cancer cells. Another study demonstrated that KIF18A is involved in the proliferation and motility of hepatocellular carcinoma (HCC) cells by regulating the cell cycle and signaling pathways linked to MMP-7/9. Given the key characteristics of *KIF18A*, we subsequently investigated the role of KIF18A in the progression of GBM.

In our study, *KIF18A* was identified as an important gene affecting the occurrence and development of GBM, suggesting that it plays an important role in glioblastoma patients, and its overexpression is related to the proliferation of cancer cells *in vivo* and *in vitro*. Therefore, our results suggest that KIF18A may be a potential therapeutic target for GBM.

## Materials and Methods

### Bioinformatic Analysis

#### Differently Expressed Genes Analysis of Young and Old Rhesus Monkeys

Paired differential expression between levels 2 and 3 and between levels 2 and 3 was investigated using edge packaging. DEG was determined using nominal significant limit *p* < 0.05 and stacking variance (FC) > 1. *p* values were tested multiple times using the Benjamini–Hochberg procedure to assess the false discovery rate (FDR). Using two network resources, DAVID (https://david.ncifcrf.gov/) and g:Profiler (https://biit.cs.ut.ee/gprofiler/), assessment of DEG (and KEGG) the richness of function classes. The *p* values of several experiments were adjusted using the Benjamini–Hochberg procedure to estimate the error detection rates (FDR).

#### Network Analysis

Using string analysis (STRING: functional protein association networks (string-db.org), we construct the network of 429 DEGs. The network was presented by Cytoscape, and the hub gene analysis in this network used the cytoHubba software.

#### Patients and Samples

In this study, 82 patients were treated according to the clinical and pathological criteria specific to the Tianjin Huanhu Hospital. All patients received written consent prior to the first surgery. The clinical and pathological characteristics of the patients were studied: age, sex, spinal tumor contusion, frequency of dementia, and ID variants. Pathology prescriptions of the tissues were examined by pathologists.

#### Immunohistochemistry Staining

Tumor specimens of glioblastoma patients were fixed with 10% v/v formalin solution, embedded in paraffin, then cut into 3-µm sections and baked at 70°C for 45 min. Following the manufacturer’s instructions and recommendations, the company used a two-step anti-displacement agent collage system (Biotin-Streptavidin HRP Detection Systems, Beijing ZSGB-BIO Technologies, Co.). Its tissues were injected with multi-shot antibodies to KIF18A (PA5-58728, Thermo Fisher Scientific; 1:250 dilution). In the laboratory test dilution rate of 1:50%, we used the tumor ratio and intensity assessment system to estimate the staining results. We used three phases of proportional assessment of the positive cell color (less than 5% of tumor-positive cells scored 0, 5–50% of tumor-positive cells scored 1, and more than 50% of tumor-positive cells scored 2). The degree of intensity of the color was also assessed by three phases (0: color or weak color, 1: color, 2: strong color). KIf18A expression was 0–2 (low) and 3-4 (high) depending on the staining intensity and percentage of positive flashes. In the absence of pathological classification and clinical information, the colored sections were independently read by two experienced pathologists.

#### Cell Culture and Transfection

U251 and U87 GBM cells were obtained from ATCC. U251 and U87 cells were maintained in ATCC-formulated Eagle’s Minimum Essential Medium (no. 30-2003), cultured with 10% fetal bovine serum and incubated from 5% CO_2_ at 37°C.

Short nucleotide sequences (shRNAs) targeting KIF18A were used to reduce expression, with cross-sequences serving as controls. Plasmids were purchased from Vigene (Cat# SH816146, Vigene Biosciences, Rockville, United States). Plasmid results from the transfection of cells with Lipofectamine^®^ 3000 (Invitrogen, Thermo Fisher Scientific, Inc.). According to the manufacturer’s protocol, 10,000 cells on 6-month-old tablets were divided into 3 groups: the sh-KIF18A group; the shControl group, after a cross-coding sequence transfer; and the sham group, which was not transferred (data not shown). Cells were collected and then subjected to quantitative PCR or IHC assays to verify transgene efficiency after 48 h. Next, the cell lines which expressed KIF18A were used in *in vitro* and *ex vivo* experiments.

#### qPCR

Total RNA is extracted using TRIzol reagents (Invitrogen) according to the instructions. Total RNA is retranscribed into the ADNC base using the synthesized M-MuLV First Strand cDNA Synthesis Kit (B532435, Sangon Biotech, China). QPCR is performed on Smart Cycler using SGExcel FastSYBR Mixture (B532954, Sangon Biotech, China). Primer sequences are in the following order: KIF18A (forward) 5′- TGC​TGG​GAA​GAC​CCA​CAC​TAT -3′, and (reverse) 5′- GCT​GGT​GTA​AAG​TAA​GTC​CAT​GA -3′; GAPDH (forward) 5′- AAC​GGA​TTT​GGT​CGT​ATT​GGG -3′, and (reverse) 5′- TCG​CTC​CTG​GAA​GAT​GGT​GAT -3′.

#### Immunoblot Assays

RIPA buffer (Cell Signaling Technology, Inc.) was used to separate GBM cells. All protein samples were extracted and lysed using Danish SDS-PAGE on PVDF membranes after lysis, and the appendix was blocked with 5% fat milk in TBST buffer. The PVDF membrane was then treated with the primary antibody for 1.5 h at room temperature. Membranes were injected with secondary antibody, and the signal was recorded for 1 h at room temperature.

#### Cell Proliferation Text

Approximately 8,000 U251 or U87 cells were seeded on 6-well culture plates, as the transfection plasmid was indicated for colony formation by incubation at 37° for 48 h. We then probed the cells in 4% PFA, stained them at 0.2% crystal violet for 30 min at room temperature, and washed them thoroughly with PBS. After that, we counted the number of colonies.

GBM cells were ejected from 96-month tablets in 48 h at 1,000 cells per hole. We treated the MT cells for 4 h, washing them twice with PBS. All stained cells were isolated using 150 ml DMSO and analyzed for OD450.

#### Tumor Growth and Metastasis Assays *In Vivo*

The care of all animals was approved by the Animal Care Committee of our hospital. To measure the tumor volume, we subcutaneously injected ksRNA-transcribed U87 cells into unstimulated mice. The tumor took 2 weeks to form, and the tumor volume was measured again. Finally, all tumors were surgically isolated and harvested for further experiments, and tumor growth curves were calculated individually.

#### Statistics

The data were analyzed with GraphPad 6.0 software. All experiments were repeated three times and were confirmed by the results obtained. Statistical comparisons were performed using Student’s *t*-test. *p* < 0.05 was considered significant. The correlation between KIF18A expression and clinicopathologic features was investigated using the χ2 criterion and * indicates *p* < 0.05.

## Results

### Identification of Kinesin Family Member 18A to Affect the Progression of Glioma as a Potential Gene

**FIGURE 1:**
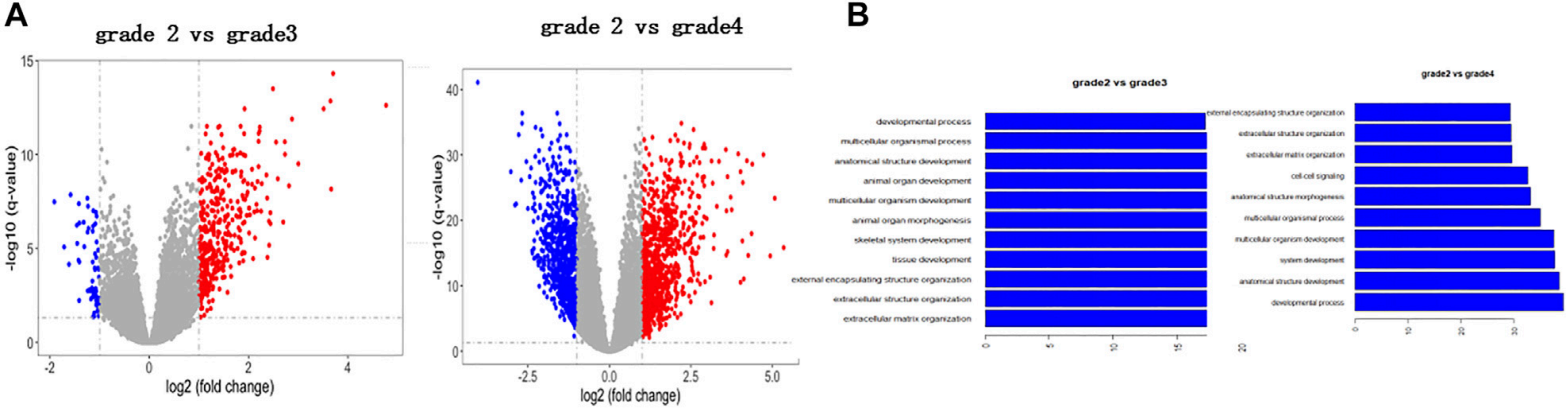
Identification of new differentially expressed genes in glioma tissues according to the bioinformatic analysis. **(A)** Volcano plot indicating changed genes in grade 2 glioma compared to grade 3 glioma. Red and blue dots indicate grade 3 glioma genes, respectively (*p* < 0.05, fold change > 2). **(B)** Volcano plot showing genetic changes in grade 2 gliomas, but not in grade 4 gliomas. Red and blue dots represent four-level glioma genes, respectively (*p* < 0.05, fold change > 2).

To explore glioma pathology, we first used bioinformatics analysis to look for potential genes that are aberrantly expressed in the glioma tissue and can influence glioma progression. First, we performed the transcriptome analysis of different stages of glioma (http://cgga.org.cn:9091/gliomasdb/) using the GLIOMASdb dataset for grade 2 gliomas, grade 3 gliomas, and gliomas grade 2. Differences in gene expression between grade 4 tumors and gliomas were analyzed. Interestingly, we found that 485 differently expressed genes (DEGs) were changed significantly as grade 2 glioma versus grade 3 glioma (Figure 1A; Supplementary Table S1), and 1883 genes were changed significantly as grade 2 glioma versus grade 4 glioma (Figure 1B; Supplementary Table S2).

**FIGURE 2:**
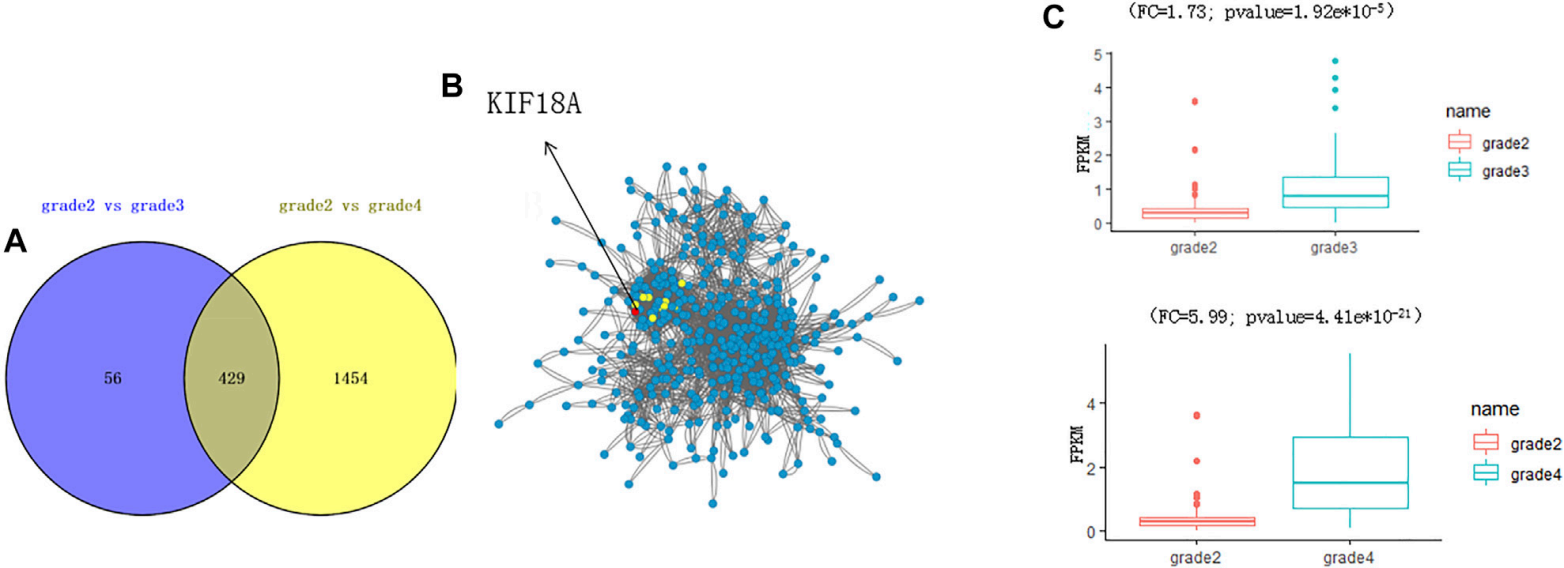
Identification of KIF18A as a potential gene to affect the progression of glioma. **(A)** The plot shows the overlapping DEGs. **(B)** The network analysis shows the interaction of 429 DEGs. The red circle shows the top five hub genes in the network, including *KIF18A*. **(C)** The boxplot shows the expression level of *KIF18A*.

We found that the biological process of DEGs was enriched by analysis of these DEGs (Figure 2A). A total of 429 genes were changed in both grade 3 and grade 4 (Figure 2B). By network analysis of 429 genes, *KIF18A* was the hub gene in the network (Figure 2C), and by informatics analysis. the expression level of *KIF18A* was upregulated in grade 3 (fold change = 1.73; adjust *p* value = 1.92e*10^−5^) and grade 4 (fold change = 5.99; adjust *p* value = 4.41e*10^−21^)) when compared to grade 2 (Figure 2C). Therefore, we assume that KIF18A is a candidate gene of glioma.

### Kinesin Family Member 18A Is Highly Expressed in Glioblastoma

**FIGURE 3:**
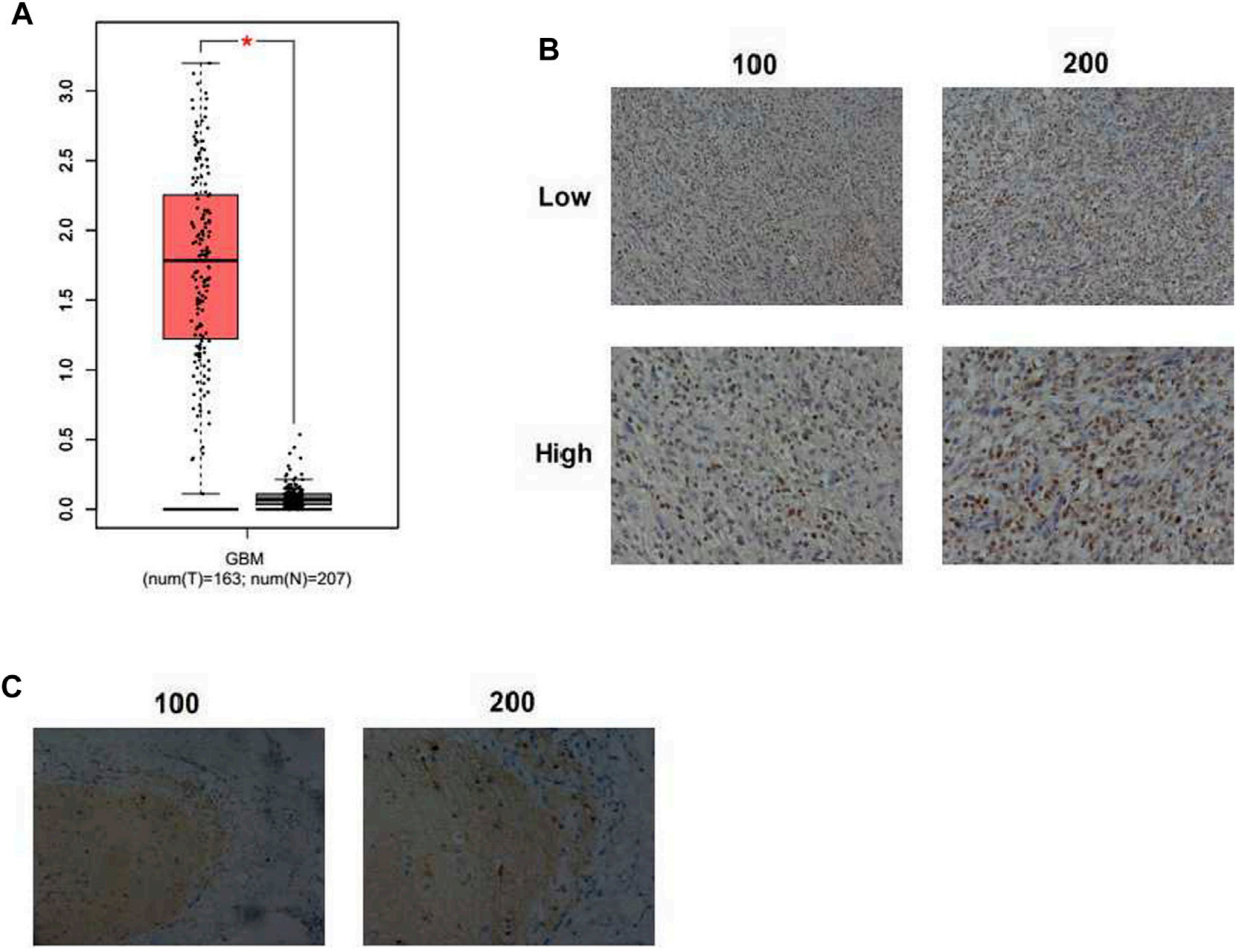
KIF18A expression in human GBM tissues. **(A)** Expression levels of KIF18A in human GBM and normal tissues (GBM: glioblastoma, *p < 0.05). **(B)** Immunohistochemical (IHC) staining of KIF18A protein in human GBM tissues (×100 and ×200 magnification, resp.). **(C)** IHC staining of KIF18A in the adjacent tissues (×100 and ×200 magnification, resp.).

To find the function of KIF18A in GBM progression, bioinformatic analysis was first conducted through an interactive web server GEPIA with the sequencing expression data of a total of 163 tumors. The mRNA expression level of KIF18A in GBM tissues was dramatically higher than that in normal tissues (*p* < 0.05, number = 163, normal tissues number = 207, resp., Figure 1A). Subsequently, the expression of KIF18A in samples removed from 82 GBM patients was detected through IHC staining. KIF18A was mainly localized in the nucleus and upregulated in GBM tissues (Figure 3B). Tumor samples were divided into two groups according to the expression of KIF18A (Figure 3B). We also noticed that KIF18A has low expression in the normal tissues (Figure 3C). These data indicated that KIF18A was highly expressed in human GBM tissues.

Subsequently, the analysis of the clinicopathological characteristics was conducted and showed the expression of KIF18A related to clinic pathological features. Moreover, the expression of KIF18A in GBM was relevant to the recurrence degree (Supplementary Table S3). It suggests that KIF18A expression is bound up with GBM progression. There was no obvious difference between high and low KIF18A groups in other clinical features, including patient age, gender, tumor lateralization, and IDH1 mutations (Supplementary Table S3).

### Kinesin Family Member 18A Promotes the Proliferation of Glioblastoma Cells *In Vitro*

Abnormal cell proliferation was known to contribute to cancer progression. We, therefore, assessed whether KIF18A could affect GBM cell proliferation and progression.

**FIGURE 4:**
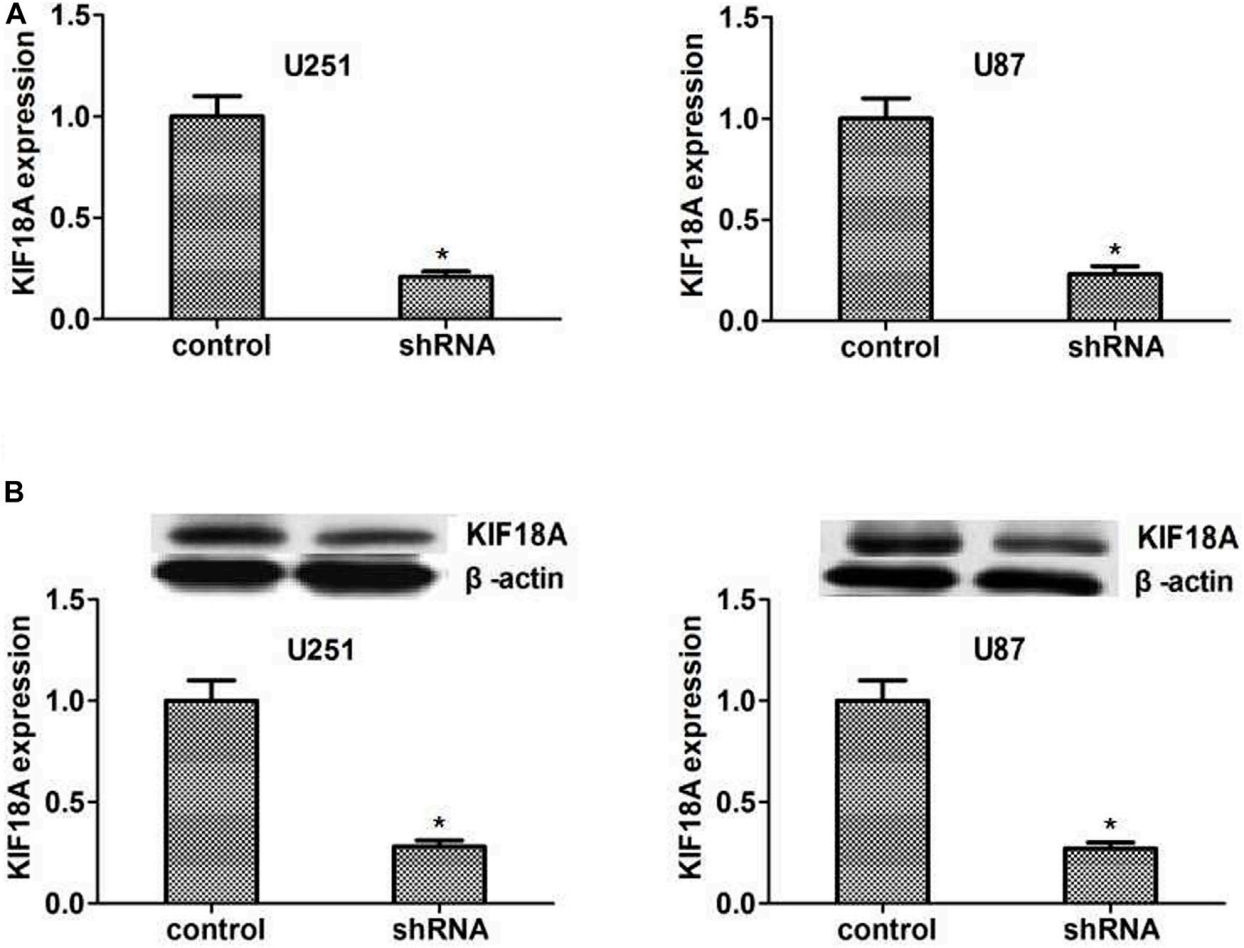
KIF18A was effectively knockdown in U251 and U87 cells caused by the shRNA transfection. **(A, B)** The qPCR assay results showed that the expression of KIF18A was significantly decreased in U251 and U87 cells, respectively. Immunoblot analysis revealed that KIF18A was significantly absent in U251 and U87 cells. Results are expressed as overall standard deviation, *p < 0.05.

We made the KIF18A stable depletion cell lines U251 and U87, respectively. Then we measured the expression of KIF18A shRNA by qPCR and Western blot. The results showed that KIF18A expression was depleted in KIF18A-shRNA-transfected U251 and U87 cells (Figures 4A,B).

**FIGURE 5:**
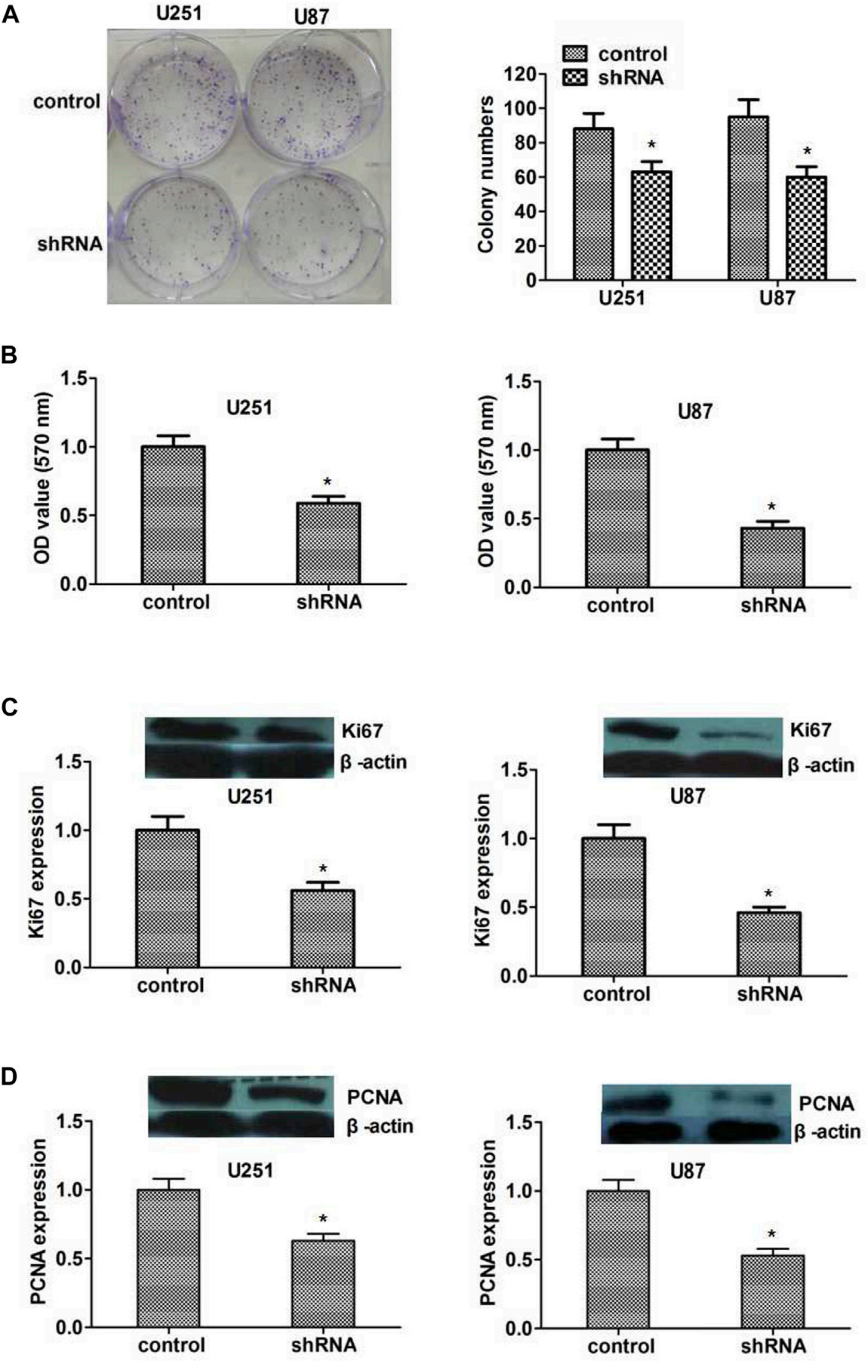
KIF18A promotes GBM cell proliferation *in vitro*. **(A)** The differences in the proliferative capacity of GBM cells after treatment. **(B)** MTT analysis shows that plasma transfer of KIF18A shRNA has an inhibitory effect on cell proliferation. **(C, D)** Immunity Blog showed that inhibition of KIF18A greatly reduced the expression of Ki67 and PCNA. Results are expressed as overall standard deviation, *p < 0.05.

Next, we investigated the effect of KIF18A on GBM cell proliferation through colony formation and MTT assays. According to the results of colony formation assays, we found that the formation capacity of the colony was significantly suppressed after KIF18A depletion, with dramatically decreased colony numbers (Figure 4A). Additionally, MTT assays provided evidence that KIF18A ablation led to a significant decrease in the absorbance value at 570 nm wavelength in U251 and U87 cells, suggesting the suppression of cell proliferation (Figure 5B). To further confirm the conclusion, we detected the expression of two key proliferation-related proteins. As expected, the expression level of Ki67 and proliferating cell nuclear antigen (PCNA) was dramatically decreased after KIF18A depletion in U251 and U87 cells (*p* < 0.05; Figures 5C,D).

In conclusion, we proved that KIF18A can be involved in GBM cell proliferation *in vitro*.

### Kinesin Family Member 18A Promotes the Tumor Growth Progression of Glioblastoma Cells in Mice

**FIGURE 6:**
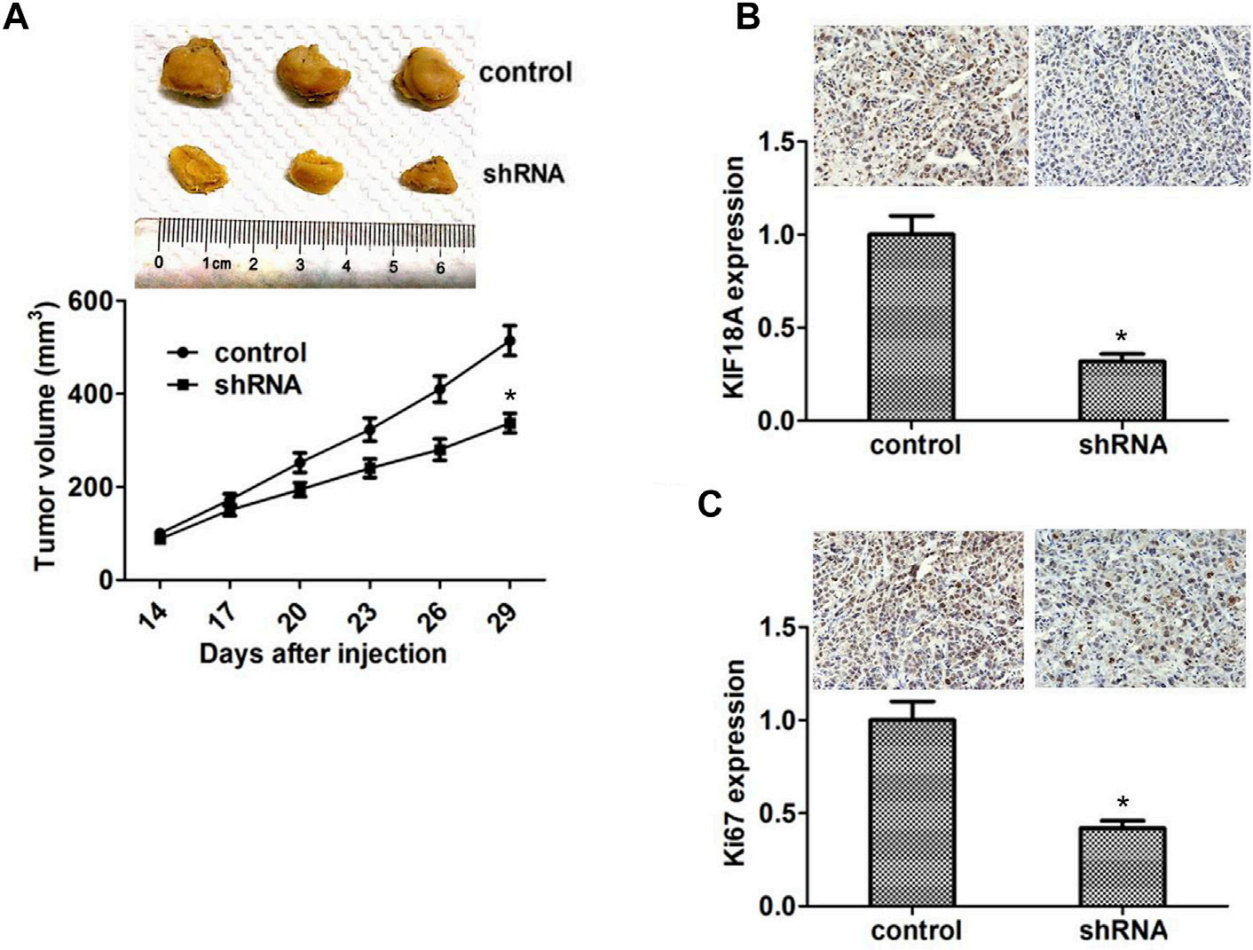
Knockdown of KIF18A impaired the tumor growth of GBM cells in mice. **(A)** U87 cells infected with KIF18A or shRNA control plasma were repopulated into nude mice. After 2 weeks, tumors were drained and tumor size was measured weekly (*n* = three groups). Tumor growth curves estimated three tumors. **(B)** Analysis shows that KIF18A is expressed in control or non-KIF18A tumor tissue from mice. **(C)** IHC analysis shows that Ki67 is expressed in control tissues or tissues lacking KIF18A. Results are expressed as overall standard deviation, *p < 0.05.

We performed xenograft assays to confirm the relationship between KIF18A expression and GBM progression. KIF18A shRNA plasmids were stably transfected with U251 cells and injected into the nude mice. After 2 weeks, tumors began to form, and volume was measured every week. We noticed that the tumor volume of the KIF18A ablation group was smaller than that of the control group (Figure 6A).

We subsequently detected the expression levels of KIF18A in tumor tissues through IHC assays. The results were that the KIF18A expression, which was compared with another, was increased in the control group (Figure 6B). In addition, we detected the expression of Ki67 in the tumor tissues through IHC assays. Consistent with the *in vitro* results, we found that the Ki67 expression was also remarkably decreased in the KIF18A depletion group (*p* < 0.05; Figure 6C). Collectively, these data showed that KIF18A promoted GBM progression *in vivo*.

## Discussion

Now, we know that the regulation of KIF18A plays an important role in many cancers (Nagahara et al., 2001; Liao et al., 2004; Zhang et al., 2010; Kasahara et al., 2016). Zhou noted that the aberrant expression of this protein is associated with the development and prognosis of hepatocellular carcinoma (Przybyl et al., 2014; Chen et al., 2017). Wozniak showed that mitosis in early tumor regulation can lead to malignancy, suggesting that overexpression of KIF18A can lead to poor control of cellular mitosis. Zhang reported that overexpression of KIF18A is associated with breast cancer metastasis and a short life span. Mismatched expression of KIF18A in MCF-7 cells leads to a multinucleus formation associated with tumor emergence. The *KIF18A* gene deletes except that it significantly suppresses tumor cell proliferation outside the body. All of the above studies suggested that tumor progression maybe needs KIF18A.

In this study, we identified that KIF18A could affect GBM progression via bioinformatic analysis. We further investigated the clinical properties and biological functions of KIF18A in glioblastoma carcinogenesis. By immunohistochemical analysis, we found that high expression of KIF18A was associated with tumor recurrence in glioblastoma patients. However, this study was a single-center and small sampled result, which should be proved further and widely. Based on the high levels of KIF18A in glioblastoma patients, we further investigated KIF18A in glioblasts using a shRNA-mediated assay. Knockout of the KIF18A gene can inhibit GBM proliferation *in vitro*. Unlike the previous studies, we could not find the effects on invasion and migration. Perhaps KIF18A might have other roles in other kinds of cancers.

To know more about the role of KIF18A in neoplasms, we studied two other proteins: Ki67 and PCNA. When KIF18A was inhabited, the expression of the two other proteins was reduced, which can promote proliferation. We injected U251 and U87 cells into nude mice. The result showed that when KIF18A was highly expressed, tumor formation was induced. However, when it expressed lower, the formation was inhabited. Kinesin motor proteins are now used in anticancer treatment (Honore et al., 2005; Marcus et al., 2005; Bhat and Setaluri, 2007; Catarinella et al., 2009; Huszar et al., 2009; Scales et al., 2017). Therefore, KIF18A could be a valuable target for the treatment of glioblastoma. Further studies of KIF18A in patients with GBM still need to clarify the exact mechanisms.

In summary, we identified *KIF18A* as a potential gene affecting GBM progression and found a correlation between the expression of KIF18A and the clinical features of GBM patients. The effects of KIF18A on GBM cell proliferation were confirmed *in vitro* and in mice. Therefore, we demonstrated that KIF18A can be a promising target in treating GBM.

## Data Availability

The original contributions presented in the study are included in the article/Supplementary Material, further inquiries can be directed to the corresponding author.

## Supplementary Material

SUPPLEMENTARY TABLE 1 The correlation analysis between KIF18A expression and the clinical features of patients with GBM.

SUPPLEMENTARY TABLE 2 Significantly expressed genes between grade 2 and grade 3 (FC > 2; adjust *p* value < 0.05).

SUPPLEMENTARY TABLE 3 Significantly expressed genes between grade 2 and grade 4 (FC > 2; adjust *p* value < 0.05).

## Abbreviations

KIF18A, kinesin family member 18A; IHC, immunohistochemical; DAB, 3,3-diaminobenzidine; HRP, horseradish peroxidase; PCNA, proliferating cell nuclear antigen; PBS, phosphate-buffered saline; PAGE, polyacrylamide gel electrophoresis; SD, standard deviation; QRT-PCR, quantificational real-time polymerase chain reaction; PI3K, phosphatidylinositol 3-kinase; shRNA, short hairpin RNA.
