# Supplementary material for: The Proliferation of Glioblastoma Is Contributed to Kinesin Family Member 18A and Medical Data Analysis of GBM
Source: Front Genet. 2022 Apr 8;13:858882. doi: 10.3389/fgene.2022.858882 (PMC9033168; doi:10.3389/fgene.2022.858882)
Supplement: Supplementary file 1 [file Table1.DOC]

**Table 1.** Relationships of KIF18A and clinicopathological characteristics in 94 patients with gliolastoma

| **Feature** | **All n=94** | **KIF18A expression** | | **χ*^2^*** | ***P*** |
| --- | --- | --- | --- | --- | --- |
|  |  | **Low** | **High** |  |  |
|  |  | **n=36** | **n=58** |  |  |
| **Age (year)** |  |  |  | 1.324 | 0.250 |
| < 55 | 54 | 18 | 36 |  |  |
| ≥ 55 | 40 | 18 | 22 |  |  |
| **Gender** |  |  |  | 0.391 | 0.532 |
| Male | 56 | 20 | 36 |  |  |
| Female | 38 | 16 | 22 |  |  |
| **Tumor lateralization** |  |  |  | 1.480 | 0.224 |
| Subtentorial | 58 | 25 | 33 |  |  |
| Supratentorial | 36 | 11 | 25 |  |  |
| **Recurrence** |  |  |  | 5.993 | 0.014* |
| Yes | 66 | 20 | 46 |  |  |
| No | 28 | 16 | 12 |  |  |
| **IDH1 mutations** |  |  |  | 0.090 | 0.764 |
| No | 53 | 21 | 32 |  |  |
| Yes | 41 | 15 | 26 |  |  |
